# The use of negative control outcomes in Mendelian randomization to detect potential population stratification

**DOI:** 10.1093/ije/dyaa288

**Published:** 2021-02-11

**Authors:** Eleanor Sanderson, Tom G Richardson, Gibran Hemani, George Davey Smith

**Affiliations:** 1 MRC Integrative Epidemiology Unit at the University of Bristol, Bristol, UK; 2 Population Health Sciences, Bristol Medical School, University of Bristol, Bristol, UK

**Keywords:** Population stratification, Mendelian randomization, negative control outcomes

## Abstract

A key assumption of Mendelian randomization (MR) analysis is that there is no association between the genetic variants used as instruments and the outcome other than through the exposure of interest. One way in which this assumption can be violated is through population stratification, which can introduce confounding of the relationship between the genetic variants and the outcome and so induce an association between them. Negative control outcomes are increasingly used to detect unobserved confounding in observational epidemiological studies. Here we consider the use of negative control outcomes in MR studies to detect confounding of the genetic variants and the exposure or outcome. As a negative control outcome in an MR study, we propose the use of phenotypes which are determined before the exposure and outcome but which are likely to be subject to the same confounding as the exposure or outcome of interest. We illustrate our method with a two-sample MR analysis of a preselected set of exposures on self-reported tanning ability and hair colour. Our results show that, of the 33 exposures considered, genome-wide association studies (GWAS) of adiposity and education-related traits are likely to be subject to population stratification that is not controlled for through adjustment, and so any MR study including these traits may be subject to bias that cannot be identified through standard pleiotropy robust methods. Negative control outcomes should therefore be used regularly in MR studies to detect potential population stratification in the data used.

## Introduction

When the observed association between an exposure, X, and an outcome, Y, is confounded by an unobserved variable, conventional regression analysis will produce misleading estimates of the effect of the exposure on the outcome. If genetic variants—usually single nucleotide polymorphisms (SNPs)—are available which reliably predict the exposure variable but do not have an effect on the outcome through any other pathway, then they are valid instrumental variables (IVs) and can be used in a Mendelian randomization (MR) analysis to obtain unconfounded evidence of the effect of the exposure on the outcome.^1^^,^^2^ A key assumption for MR to give consistent estimates of the causal effect of an exposure on the outcome is that the SNPs used as instruments are not associated with the outcome other than through the exposure.^3^ One way in which this assumption may be violated is through population stratification, where structure in the population studied causes a correlation at the population level between the distribution of the genetic variants and the distribution of the exposure and/or outcome.^4^^,^^5^ Population stratification and its implications for genome-wide association studies (GWAS) are described in more detail in Box 1. MR analyses are often conducted by comparing summary data estimates of SNP-exposure and SNP-outcome associations gleaned from two independent but homogeneous study populations. This is referred to as two-sample summary data MR.^6^ For the MR estimate of the causal effect of the exposure on the outcome to be a consistent estimate of the effect of the exposure on the outcome, the genetic variants must satisfy the following assumptions.

**Box 1. Population Stratification and the effects on results from GWAS**

 

Population stratification occurs where different sub-populations within a population being studied have different distributions of both allele frequencies and the phenotype being considered. Phenotypes, particular social phenotypes, have different distributions geographically, even over relatively small geographical areas. Primarily due to genetic drift, allele frequencies will also differ geographically. Even if this distribution of allele frequencies occurs due to chance, it can lead to an apparent association between those alleles and the phenotype, even when none exists. This will confound the results from the GWAS and lead to potentially spurious or inflated association between SNPs and the phenotype, which are due to the structure of the population and not due to a direct effect of the SNP on the phenotype.^2^^,^^18^ Additionally it means that even for SNPs that do have a direct effect on the phenotype, the estimated size of that association will be biased.^19^

Within GWAS studies, population stratification is often controlled for by adjusting for the top principal components from a principal components analysis of the genetic variants,^20^ or by using linear mixed models which allows them to account for genetic confounding of common variants more accurately, and improve power, by jointly modelling the contribution of all measured variants.^21–23^ However, a number of recent papers have examined the effect of population stratification in datasets such as UK Biobank and have shown that population stratification is likely to present challenges for causal inference and that such adjustment may not be sufficient.^5^ Haworth and colleagues show in UK Biobank that genetic variants are associated with a number of variables including location of birth, and this association cannot be fully accounted for by standard principal components analysis.^4^ Abdellaoui and colleagues show that many traits in UK Biobank are subject to genetically driven clustering after controlling for ancestry.^24^ They propose that this clustering is likely to reflect socioeconomic differences in migration patterns, and that these results suggest that social stratification affects the geographical pattern of allele frequencies. The implication of this is that even very recent patterns of movement within the UK will lead to population stratification for more ‘social’ phenotypes.

Many GWAS are conducted using multiple independent cohorts, each assumed to have independent ancestral patterns that are likely to be cancelled out when meta-analysed. A recent study has brought this assumption into question, illustrating that within-cohort correction for population stratification tends to be under-powered to fully account for deep ancestral history that is common across all cohorts.^25^

Two recent papers show that a polygenic signal for height, observed in European GWAS such as GIANT, is weak or absent in UK Biobank and that the signal observed in the European GWAS is due to population stratification.^26^^,^^27^ Barton and colleagues highlight why this matters. When multiple genetic variants are used to predict a phenotype, as is the case in an MR study, the association between each variant and the phenotype needs to be unbiased for reliable inference. Population stratification will bias these associations and therefore potentially any inference that is based on them.^19^

IV1: the variants must be associated with the exposure X (the ‘relevance’ assumption).
IV2: the variants must be independent of all (observed or unobserved) confounders of X and Y, as represented by U (the ‘exchangeability’ assumption).
IV3: the variants must be independent of the outcome Y given the exposure X, (the ‘exclusion restriction’).

**Figure 1:**
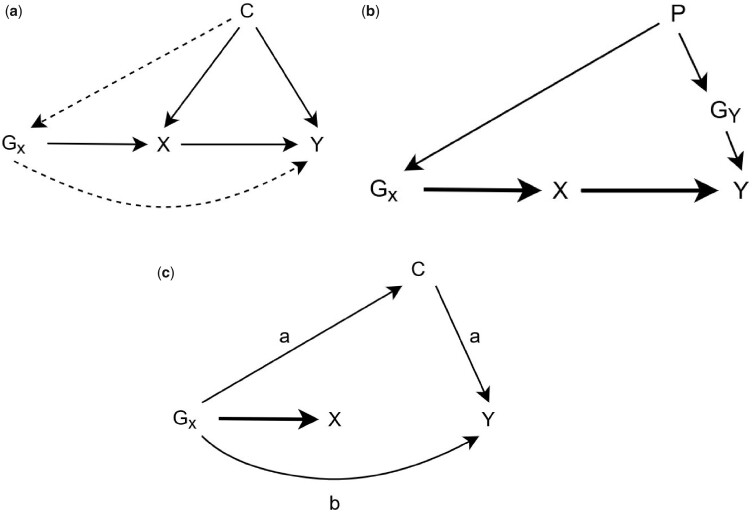
Instrumental variable assumptions, and violation of these assumptions through population stratification or pleiotropy. (a) Instrumental variable assumptions. (b) Confounding of the genetic instrument and outcome introduced by population stratification. (c) Mechanisms through which pleiotropy can cause bias in Mendelian randomization estimates. X is the exposure of interest, Y is the outcome of interest, G_x_ are the genetic variants associated with X used as instruments, G_Y_ are genetic variants associated with Y, C is a confounder of the exposure outcome relationship. In (a): assumption IV1 is illustrated by the bold line from G_X_ to X. Violations of assumptions IV2 and IV3 are given by the dashed lines from C to G_X_ and from G_X_ to Y, respectively. In (b): the presence of population stratification creates an association between G_X_ and Y that does not go through X, violating one of the IV assumptions. In (c): pleiotropy will cause bias in MR estimates if either both edges marked a, or the edge marked b, are present. Pleiotropy in MR studies is explained in detail elsewhere^3^

These assumptions are illustrated in Figure 1 and are explained in detail elsewhere.^3^^,^^6^^,^^7^

Population stratification causes confounding of the instrument and outcome in MR, violating IV2, illustrated in Figure 1. Any MR analyses based on the results from a GWAS will potentially be biased if that GWAS does not fully account for any ancestral population structure that could lead to population stratification.^5^^,^^8^ This bias is likely to be largest when the outcome phenotype in an MR study is subject to population stratification that has not been fully accounted for. However, it will also bias effect estimates in a two-sample MR analysis when the exposure phenotype is subject to population stratification, by causing the estimated association between the SNP and the exposure to be mis-specified. As well as increasing or decreasing the size of the observed association, this bias could generate evidence of an apparent causal effect of the exposure on the outcome when no such effect exists, or alternatively could mask a true effect.

A method that is often used in observational studies to detect confounding, and help assessment of whether a causal relationship exists between an exposure and an outcome, is negative control outcome analysis.^9–11^ ‘Negative controls’ essentially renames the ‘specificity of associations’ which Hill considered a factor that should be weighed up in evaluating plausibility of causation in epidemiological studies.^12^ Given the dismissal of this criterion in some influential epidemiological texts, their rechristening as ‘negative controls’ allowed their relegitimation.^13^ Negative control outcome studies compare the association observed between an exposure and the outcome under investigation with the association observed between that exposure and a negative control outcome. The negative control outcome variable is chosen to be a variable that is not expected to be associated with the exposure of interest, but is expected to be subject to the same unobserved confounding as the exposure and outcome of interest. It follows that if the assumptions hold, any association observed between the exposure and the negative control outcome will be due to confounding in the model. Negative control outcome studies have previously also been proposed to detect selection bias in observational studies.^11^ We advance the use of negative control outcomes to identify when exposure and outcome phenotypes in an MR analysis may be subject to population stratification or selection bias that has not been fully accounted for in the GWAS, and so may induce instrument-outcome confounding or mis-estimation of the SNP exposure relationship, and consequently may bias the results obtained.

Negative control outcome methods applied to an MR analysis have been used in a limited number of studies previously to detect potential pleiotropy and provide additional evidence on the validity of the MR study.^14–16^ These negative controls were, however, not used in the detection of potential population stratification. We illustrate this method through estimation of the potential for population stratification in 33 preselected exposure-indexing GWAS included in MR Base.^17^ We detect potential population stratification by estimating the effect of each phenotype on self-reported tanning ability and self-reported natural hair colour, variables that are likely to be highly affected by population stratification but that are largely determined at birth and are not expected to be truly affected by any of the phenotypes considered. Our results from this study show that the GWAS of adiposity-related phenotypes and education are likely to be affected by population stratification. Any MR involving these phenotypes is therefore potentially subject to bias. These results show that negative control outcomes should be routinely used in MR studies to detect population stratification.

## Methods

The GWAS of natural hair colour was conducted using data from UK Biobank [https://www.ukbiobank.ac.uk/] under application number 15825. UK Biobank has received ethical approval from the UK National Health Service’s National Research Ethics Service (ref 11/NW/0382). All other analyses were conducted using publicly available summary data generated using the relevant ethics approval for that study.

Two-sample summary data MR compares the association of a set of SNPs with the exposure and outcome to determine the effect of the exposure on the outcome. It is explained in detail elsewhere.^6^^,^^7^ We propose running two additional MR sensitivity analyses for any MR study where population stratification is thought to potentially affect the result:

- an MR analysis to estimate the effect of the exposure of interest on the negative control outcome;
- an MR analysis to estimate the effect of the outcome of interest on the negative control outcome (i.e. the outcome of interest becomes the exposure in this analysis).

Any effects detected in these analyses would indicate the potential presence of population stratification in the GWAS of the phenotype of interest and therefore possible bias in an MR analysis including that phenotype. The negative control outcomes should be selected based on the same criteria that have been traditionally used in epidemiological studies; i.e. they should not be expected to be dependent on the phenotypes of interest in the analysis but should be affected by the same confounding. In order to satisfy the assumption that the negative control outcome is not actually caused by the exposure, we propose using phenotypes that are determined before the exposure and the outcome in the negative control MR study. The phenotype for the negative control outcome should also be selected to be thought to be affected by the population stratification. For bias caused by population stratification, such variables could include hair colour, eye colour or skin tone. If there is no instrument-outcome confounding, this analysis will give a null result. As the negative control outcomes are largely predetermined relative to the exposure and outcome and so cannot depend on either, any association of the SNPs with the negative control outcome must be driven by some other mechanism. This could take the form of pleiotropy due to the SNPs having an effect either directly on the negative control outcome or on another phenotype that then affected the negative control outcome, illustrated in Figure 1. However, conventional pleiotropy robust estimation methods will give results that are robust to this pleiotropy if it only affects some of the SNPs included in the estimation.^28–30^ Alternatively, the observed effect of the phenotype on the negative control outcome could be due to instrument -outcome confounding. In this case, conventional pleiotropy robust methods would not give results that are robust to this bias as the confounding would affect all of the SNPs included in the estimation. Evidence of an effect of the exposure and outcome on the negative control outcome indicates that an MR study of the exposure on the outcome is also likely to be biased. An illustration of how negative control outcomes could be applied to an MR estimation of body mass index on coronary heart disease is given in Supplementary Figure S1, available as Supplementary data at *IJE* online.

## Applied example

To illustrate the use of negative control outcomes in MR studies, we investigated the effect of a range of exposures on self-reported tanning ability and natural hair colour from UK Biobank, as a negative control outcome in two-sample summary data MR to detect population stratification in these exposures. Between 2006 to 2010, the UK Biobank study enrolled 500 000 individuals aged between 40 and 69 at baseline across 22 assessments centres in the UK.^31^ Data were collected based on clinical examinations, assays of biological samples, detailed information regarding self-reported health characteristics and genome-wide genotyping.^32^ In total, 12 370 749 genetic variants in up to 463 005 individuals were available for analysis, as described previously.^33^ UK Biobank received ethical approval from the Research Ethics Committee (REC reference for UK Biobank is 11/NW/0382).

For their tanning response to sun exposures, individuals were asked ‘What would happen to your skin if it was repeatedly exposed to bright sunlight without any protection?,’ with four potential responses which ranged from get very tanned (given a score of 1) to never tan and always burn (given a score of 4). A higher score is therefore associated with fairer skin that is less prone to tanning. A GWAS of this question was conducted by the MRC IEU^33^ and included in MR Base.^17^ For natural hair colour, individuals were asked ‘What best describes your natural hair colour? (If your hair colour is grey, the colour before you went grey)?’, with five valid potential responses; blonde, red, light brown, dark brown or black. We categorized these responses as 1: blonde, 2: red, 3: light brown, 4: dark brown and 5: black, in accordance with a previous GWAS of hair colour which included UK Biobank.^34^ The association between genetic variants and outcomes in the UK Biobank study were assessed using the software BOLT-LMM.^21^^,^^33^ This approach applies a Bayesian linear mixed model to evaluate the association between each genetic variant across the human genome in turn, with the analysed outcome accounting for both relatedness and population stratification.^22^ Age at baseline, sex and type of genotyping array were added as covariates in the model. As tanning ability and hair colour are largely determined at birth and are highly dependent on variations in an individual’s ancestral background, they should not depend on exposures experienced during an individual’s lifetime.

We preselected 50 characteristics or risk factors with GWAS data available in MR base as our example phenotypes. These phenotypes were all selected to have male and female participants from a mixed or European population that did not include UK Biobank. Where multiple GWAS for the same phenotype were available, we chose only the most recent relevant one available in MR base at the time of analysis; however, we retained in the analysis similar (but not exactly equivalent) phenotypes such as body mass index (BMI) and waist-to-hip ratio. We excluded GWAS that included UK Biobank to avoid the potential for winner’s curse from selecting the exposure and the outcome from the same sample. A full list of the phenotypes included in the analysis is given in Supplementary Table S1, available as Supplementary data at *IJE* online. From these preselected phenotypes, we excluded one GWAS due to the information available in MR base not matching that given in the paper, and 16 with fewer than five genome-wide significant SNPs available as instruments, leaving us with 33 phenotypes for analysis.

For each of our 33 exposures, we calculated the inverse variance weighted (IVW) effect for that exposure on tanning ability and hair colour. For those exposures which showed evidence of an effect on each negative control outcome, we also report the MR Egger,^30^ weighted mode^29^ and weighted median^28^ effects as sensitivity analyses. Weighted mode and median estimates give robust estimation results if the association observed is driven by outlying SNPs. However, if population stratification is driving the results seen, this would not be expected to be due to an effect of a small number of outlying SNPs but due to an effect across all of the SNPs used as instruments. We would therefore still expect to estimate an effect of the trait on the negative control outcome in each case. MR Egger accounts for violation of IV assumptions 2 and 3 that satisfy the InSIDE assumption. This assumption states that the bias on the outcome is independent of the strength of the SNP on the exposure. Bias due to population stratification may satisfy this assumption if it applies equally across the SNPs. However, MR Egger has low power to detect effect estimates, and so it is often not possible to determine whether the lack of an association in an MR Egger estimation that was observed in an IVW analysis is due to bias in the IVW estimation or low power in the MR Egger estimates. In each case we included all SNPs that are genome-wide significant for the exposure as our genetic instruments, as these are the SNPs that are usually used for MR analyses with multiple SNPs.^35^ All analyses were conducted using the package ‘TwoSampleMR’ in R.^17^

**Figure 2:**
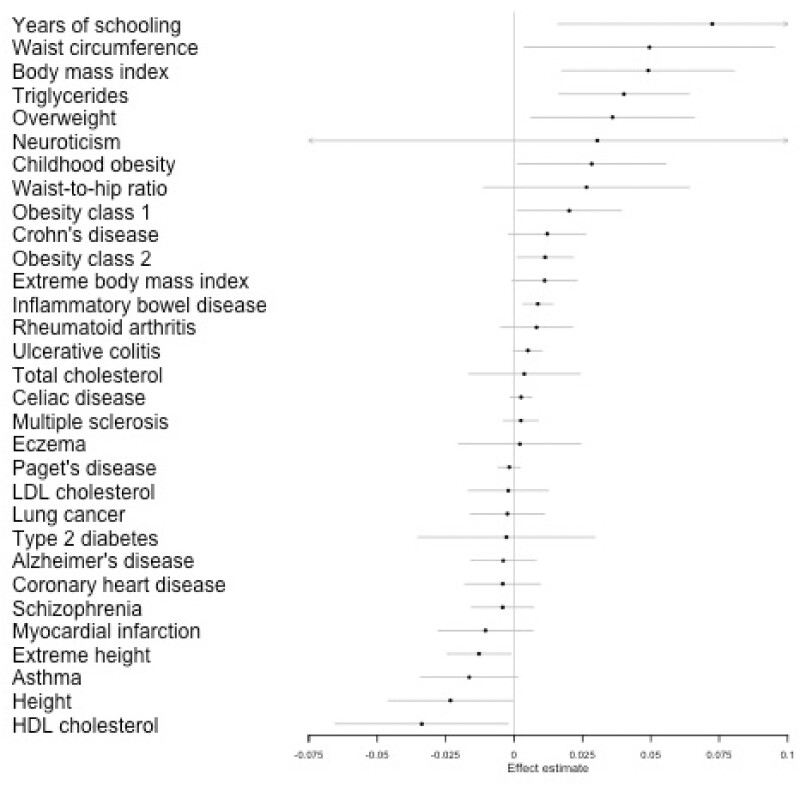
Inverse variance weight (IVW) estimates from Mendelian randomization (MR) analyses on self-reported tanning ability. IVW results from MR analyses of 33 preselected traits on tanning ability. A higher score indicates less being less likely to tan and more likely to burn when exposed to strong sunlight. Full results from these analyses are given in Supplementary Table S1

Estimated effects of the genetic liability towards each exposure on tanning ability from our IVW analysis are given in Figure 2, with full details given in Supplementary Table S1. These results show that a number of the exposures considered appear to have a causal effect of genetic liability towards that exposure on tanning ability. Table 1 gives the estimated effect sizes for all results with a *P*-value of less than 0.05 in the IVW analyses. Although we have conducted multiple tests in this analysis as many of the phenotypes we consider are related, these tests are not independent. We therefore suggest here that this gives a potential indication of whether results warrant further investigation for potential bias rather than a hard cut-off for whether these results are of interest. The traits with an effect on tanning fall into three categories; adiposity-related traits, bowel disease and years of schooling. The majority of the GWAS studies included adjusted for population stratification (using principal components or alternative methods), suggesting that this adjustment alone is not sufficient to remove all structural bias in the data. The MR Egger results suffer from high levels of uncertainty due to low power but estimated the same direction of effect in all but three of these exposures. The weighted mode and weighted median estimates supported the overall results with all of the results showing the same direction of effect as the IVW results and only three of the 12 results not replicating in at least one of the weighted mode or weighted median estimates.

**Table 1:** Full Mendelian randomization (MR) results for exposures which show potential association with tanning ability

Exposure	No. SNPs	Est. method	Effect	Std error	*P*-value	95% confidence interval	
Years of schooling	68	IVW	0.072	0.029	0.012	[0.016	0.104]
		MR Egger	0.056	0.149	0.710	[-0.237	-0.409]
		Weighted median	0.092	0.026	<0.001	[0.040	0.171]
		Weighted mode	0.103	0.049	0.038	[0.007	0.118]
Childhood obesity	5	IVW	0.028	0.014	0.040	[0.001	0.031]
		MR Egger	−0.088	0.100	0.444	[-0.285	−0.646]
		Weighted median	0.018	0.007	0.009	[0.004	0.026]
		Weighted mode	0.015	0.008	0.109	[0.001	0.017]
Body mass index	78	IVW	0.049	0.016	0.002	[0.018	0.083]
		MR Egger	0.055	0.039	0.165	[-0.022	0.012]
		Weighted median	0.047	0.018	0.008	[0.012	0.071]
		Weighted mode	0.038	0.019	0.050	[0.001	0.039]
HDL cholesterol	84	IVW	−0.034	0.016	0.036	[-0.065	−0.162]
		MR Egger	−0.006	0.030	0.848	[-0.064	−0.131]
		Weighted median	−0.020	0.010	0.046	[-0.040	−0.100]
		Weighted mode	−0.019	0.009	0.030	[-0.037	−0.091]
Triglycerides	55	IVW	0.040	0.012	0.001	[0.016	0.072]
		MR Egger	0.024	0.019	0.226	[-0.014	−0.004]
		Weighted median	0.014	0.011	0.226	[-0.008	−0.003]
		Weighted mode	0.010	0.012	0.406	[-0.014	−0.017]
Inflammatory bowel disease	62	IVW	0.009	0.003	0.002	[0.003	0.015]
		MR Egger	0.008	0.007	0.273	[-0.006	−0.004]
		Weighted median	0.004	0.003	0.185	[-0.002	0.000]
		Weighted mode	0.003	0.004	0.459	[-0.005	−0.007]
Waist circumference	45	IVW	0.050	0.023	0.033	[0.004	0.057]
		MR Egger	0.098	0.062	0.121	[-0.023	0.052]
		Weighted median	0.052	0.022	0.017	[0.009	0.070]
		Weighted mode	0.035	0.022	0.128	[-0.009	0.017]
Extreme height	44	IVW	−0.013	0.006	0.031	[-0.024	−0.061]
		MR Egger	−0.044	0.027	0.115	[-0.097	−0.234]
		Weighted median	−0.003	0.003	0.257	[-0.009	−0.021]
		Weighted mode	0.000	0.006	0.932	[-0.011	−0.023]
Obesity class 1	17	IVW	0.020	0.010	0.038	[0.001	0.022]
		MR Egger	−0.009	0.027	0.735	[-0.063	−0.133]
		Weighted median	0.014	0.007	0.042	[0.001	0.015]
		Weighted mode	0.013	0.007	0.100	[-0.002	0.010]
Obesity class 2	11	IVW	0.011	0.005	0.030	[0.001	0.014]
		MR Egger	0.002	0.016	0.927	[-0.030	−0.058]
		Weighted median	0.012	0.006	0.026	[0.001	0.015]
		Weighted mode	0.011	0.006	0.099	[-0.001	0.009]
Overweight	14	IVW	0.036	0.015	0.018	[0.006	0.048]
		MR Egger	−0.025	0.050	0.630	[-0.124	−0.267]
		Weighted median	0.028	0.011	0.011	[0.006	0.040]
		Weighted mode	0.029	0.012	0.033	[0.005	0.039]

Results from inverse variance weight (IVW), MR Egger, weighted mode and weighted median analyses for those phenotypes which indicated a potential effect on tanning ability from an MR analysis of 33 preselected phenotypes on tanning ability.

SNPs, single nucleotide polymorphisms; Est., estimation; Std, standard; HDL, high-density lipoprotein.

**Figure 3:**
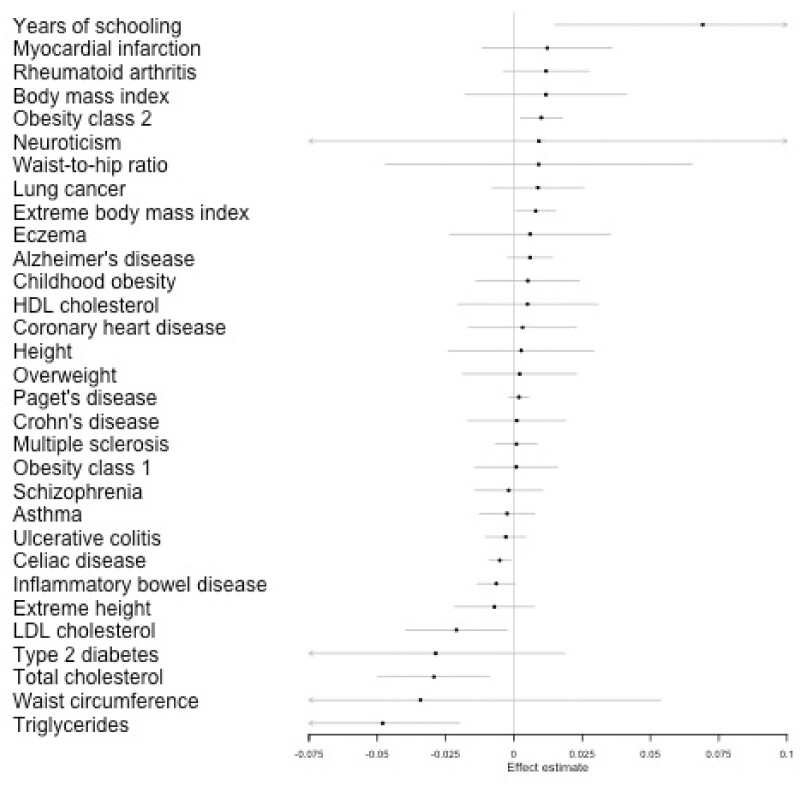
Inverse variance weight (IVW) estimates from Mendelian randomization (MR) analyses on self-reported natural hair colour. IVW results from MR analyses of 33 preselected traits on hair colour. A higher score indicates darker hair colour. Full results from these analyses are given in Supplementary Table S1

Estimated effects of the genetic liability towards each exposure on hair colour from our IVW analysis are given in Figure 3, with full details given in Supplementary Table S1. Results from the IVW analyses and sensitivity analyses for exposures with a *P*-value <0.05 (as a heuristic for presentation) in the IVW analysis are given in Table 2. These results show a very similar pattern to the results for tanning ability with adiposity- and education-related traits, showing a potential effect on hair colour. Additionally, coeliac disease showed an association with hair colour. Three traits (triglycerides, years of schooling and obesity class 2) showed evidence of an effect on both tanning ability and hair colour.

**Table 2:** Mendelian randomization (MR) results for exposures which show potential association with hair colour

Exposure	No. SNPs	Est. method	Effect	Std. error	*P*-value	95% confidence interval	
Years of schooling	71	IVW	0.070	0.027	0.012	[0.017	0.123]
		MR Egger	0.069	0.140	0.502	[-0.205	0.342]
		Weighted median	0.074	0.024	0.002	[0.027	0.120]
		Weighted mode	0.112	0.060	0.046	[-0.005	0.230]
Coeliac disease	13	IVW	−0.004	0.002	0.012	[-0.008	0.000]
		MR Egger	−0.007	0.003	0.010	[-0.012	−0.002]
		Weighted median	−0.007	0.002	0.005	[-0.011	−0.002]
		Weighted mode	−0.005	0.002	0.002	[-0.009	−0.001]
LDL cholesterol	79	IVW	−0.020	0.009	0.025	[-0.038	−0.002]
		MR Egger	−0.016	0.013	0.189	[-0.042	0.010]
		Weighted median	−0.025	0.008	0.001	[-0.040	−0.010]
		Weighted mode	−0.020	0.006	<0.001	[-0.031	−0.009]
Total cholesterol	87	IVW	−0.028	0.010	0.005	[-0.048	−0.009]
		MR Egger	−0.018	0.016	0.212	[-0.050	0.014]
		Weighted median	−0.026	0.008	0.003	[-0.043	−0.009]
		Weighted mode	−0.026	0.007	<0.001	[-0.040	−0.012]
Triglycerides	54	IVW	−0.053	0.015	0.001	[-0.082	−0.024]
		MR Egger	−0.059	0.025	0.044	[-0.107	−0.010]
		Weighted median	−0.032	0.012	0.008	[-0.054	−0.009]
		Weighted mode	−0.037	0.013	0.040	[-0.063	−0.011]
Extreme body mass index	7	IVW	0.008	0.004	0.028	[0.001	0.015]
		MR Egger	−0.017	0.017	0.375	[-0.050	0.017]
		Weighted median	0.006	0.005	0.173	[-0.003	0.016]
		Weighted mode	0.001	0.006	0.812	[-0.010	0.013]
Obesity class 2	11	IVW	0.010	0.004	0.010	[0.002	0.018]
		MR Egger	−0.011	0.012	0.353	[-0.034	0.012]
		Weighted median	0.004	0.005	0.427	[-0.006	0.015]
		Weighted mode	0.002	0.006	0.710	[-0.009	0.014]

Results from inverse variance weight (IVW), MR Egger, weighted mode and weighted median analyses for those phenotypes which indicated a potential effect on tanning ability from an MR analysis of 33 preselected phenotypes on self-reported natural hair colour. *P*-values in parentheses.

SNPs, single nucleotide polymorphisms; Est., estimation; Std, standard; LDL, low-density lipoprotein.

These results illustrate the use of negative control outcomes to detect potential population stratification, and show that the GWAS results for many of the phenotypes we consider, particularly those related to education and adiposity, are likely to be affected by population stratification.

## Discussion

In this paper we describe the use of variables that are predetermined relative to the phenotypes of interest, but are likely to be subject to population stratification as negative control outcomes within an MR analysis to detect population stratification. The method we describe is easy to implement with currently available software and data. Our results suggest that negative control outcomes could be routinely used as part of any MR study, to detect population stratification in GWAS data that could bias the results from the MR estimation.

We propose using this method to examine the potential for population stratification in both the exposure and outcome in any MR study. Population stratification in the outcome can create confounding between the genetic variants and the outcome, which can lead to an apparent association between an exposure and outcome in any MR estimation when no causal effect exists. Population stratification in the exposure will create confounding of the genetic variants and exposure which can bias the causal estimate obtained from the MR estimation, including making a true association appear to be null. Therefore, for reliable interpretation of the results from the MR estimation, including a reliable assessment of whether or not a causal effect exists as well as estimation of the size of that effect, it is important that population stratification does not affect either the exposure or outcome. For this reason, negative control outcomes should be applied as described here to both the exposure and the outcome in the MR estimation of interest.

Our applied analysis conducts a negative control outcome MR analysis of 33 preselected phenotypes on tanning ability and natural hair colour, to detect the potential for population stratification in the GWAS of these phenotypes. We find a range of phenotypes are potentially affected by population stratification, particularly a number of phenotypes related to BMI, height and educational attainment. Any association between these variables shown by an MR analysis could be due to population stratification introducing the apparent association. This result is supported by a recent study using within-family MR analyses, which showed the observed associations from MR analyses between height and education and BMI and education attenuated once family effects were controlled for.^8^ One key advantage of within-family MR over our method is that is can provide MR causal estimates adjusted for the bias due to population stratification. However, within-family MR requires a large sample of related individuals and cannot be conducted with standard GWAS results. The method we propose can detect potential population stratification in samples that do not contain related individuals and using existing summary data.

Genetic liability for coeliac disease was associated with hair colour; however, this GWAS did not account for population stratification, suggesting that the adjustments for population stratification included in GWAS studies do mitigate the effects of population stratification to some degree. However, a number of the other exposures which were found to be associated with our negative control outcomes did include adjustment for population stratification in the GWAS, suggesting that this adjustment does not fully mitigate the effects. Examination of the extent to which adjustment for population stratification, through inclusion of principal components or alternative adjustments such as using a BOLT-LMM model^21^ in GWAS, can mitigate the problems of bias due to instrument-outcome confounding in MR studies, is an area for future research.

Linkage disequilibrium (LD) score regression is a method that attempts to separate out biological and confounded genetic signals and so can also be applied in an MR setting to determine whether there is a causal (biological) effect of an exposure on an outcome or if an observed association is due to confounding.^36^ LD score regression however does not give the appropriate results if the GWAS being considered have been performed using a linear mixed model. Our method therefore provides a complementary approach to LD score regression whcih does not depend on the method used to estimate GWAS associations. Additionally, LD score regression incorporates data from the entire genome, whereas the use of negative controls outcomes proposed here only uses SNPs strongly associated with the phenotypes of interest. This is potentially more relevant to bias in MR analyses which use SNPs associated with the exposure to estimate the causal effect of the exposure on the outcome.

The methods described here could equally be applied to detect selection bias. Selection bias, where individuals select to participate in a study or not, based on their particular phenotypes, can also induce bias into any analyses of that study.^37^ Particularly, selection bias can induce bias in the associations observed between phenotypes selected on and the genetic variants associated with those phenotypes.^38^^,^^39^ Selection bias is a form of collider bias which occurs when the variables of interest independently affect a third variable and so conditioning on this third collider variable will induce an association between the variables of interest.^37^^,^^40^ In this case, the third variable is participation in the study and conditioning on it is unavoidable, as data are only available for the participants. Although selection bias is distinct from population stratification in its source, the subsequent biases in MR studies are similar.^39^^,^^41^ Negative control outcomes could also be used to detect selection bias in GWAS results by using outcome phenotypes that are expected to affect participation in a study, but that are predetermined relative to the exposure and outcome considered in the MR analysis. Such negative control outcomes could include early life variables such as place of birth or education. Alternatively, participation can be examined directly in birth cohort studies which are followed up over time, and GWAS results from these studies could be used as a negative control outcome.^42^

There are a number of weaknesses with our method that should be considered. This method is only able to detect bias as far as it affects the chosen negative control outcome, and therefore no detected effect of the phenotype on the negative control outcome does not mean that the phenotype, and any associated MR analysis, is necessarily free from bias. This limitation can be mitigated by choosing negative control outcomes that are likely to be highly population stratified, as far as they are available.

Negative control outcome calibration has been proposed for observational negative control studies, to adjust the effect of the exposure of interest on the outcome for the bias detected by the negative control outcome.^43^ We believe that mechanical application of such an approach should be avoided due to the strong assumptions required for such calibration to give reliable estimates.^44^ A key assumption for such an approach to work is that the model fully identifies the effect of the exposure on the outcome and negative control outcome, such that the size of the effect of the bias on the outcome can be determined once and differences in scale of the outcome and negative control have been taken into account. In the context of MR this is not a reasonable assumption, as this assumption would require the instrument-outcome confounding to have exactly the same effect in the exposure, outcome and negative control outcome, and so if bias is detected, this method does provide a method to correct the estimated effect. However, the size and direction of the estimated effect on the negative control outcome could be used as an indicator for a sensitivity analysis which considered whether bias of up to, for example, five times that estimated by the negative control outcome would change the conclusions from the main MR analyses.

An extension to this method is to consider the use of similar phenotypes, considered as negative control outcomes here, as negative control exposures. Such an approach provides an obvious complement to the approach considered here; however, the assumptions required and implications of such an analysis are notably different from those for a negative control outcome study, and therefore we leave this as an area for future research.

## Supplementary data

Supplementary data are available at *IJE* online.

## Funding

This work was supported by the Integrative Epidemiology Unit which is funded by the University of Bristol and the Medical Research Council (MC_UU_00011/1). T.G.R. is a UKRI Innovation Research Fellow (MR/S003886/1).

## Data availability

Data for all outcomes considered and ‘tanning response to sun exposure’ exposure are available as part of the R package ‘TwoSampleMR’. Code for the negative control analyses conducted is available at [https://github.com/eleanorsanderson/MR-negativecontrols].

## Author contributions

E.S. and G.D.S. conceived of the study. T.G.R. conducted the GWAS of hair colour. E.S. conducted all other analyses and wrote the first draft of the manuscript. All authors edited and approved the manuscript.

## Conflict of interest

None declared.

## Supplementary Material

dyaa288_Supplementary_Data

## References

[1] Davey Smith G, Ebrahim S. ‘Mendelian randomization’: can genetic epidemiology contribute to understanding environmental determinants of disease? Int J Epidemiol 2003;32:1–22. 12689998 10.1093/ije/dyg070

[2] Lawlor DA, Harbord RM, Sterne JAC, Timpson N, Davey Smith G. Mendelian randomization: Using genes as instruments for making causal inferences in epidemiology. Stat Med 2008;27:1133–63. 17886233 10.1002/sim.3034

[3] Hemani G, Bowden J, Davey Smith G. Evaluating the potential role of pleiotropy in Mendelian randomization studies. Hum Mol Genet 2018;27:R195–R208. 29771313 10.1093/hmg/ddy163 PMC6061876

[4] Haworth S, Mitchell R, Corbin L et al Apparent latent structure within the UK Biobank sample has implications for epidemiological analysis. Nat Commun 2019;10:333. 30659178 10.1038/s41467-018-08219-1 PMC6338768

[5] Lawson DJ, Davies NM, Haworth S et al Is population structure in the genetic biobank era irrelevant, a challenge, or an opportunity? Hum Genet 2020;139:23–41. 31030318 10.1007/s00439-019-02014-8 PMC6942007

[6] Burgess S, Scott RA, Timpson NJ, Davey Smith G, Thompson SG; EPIC-InterAct Consortium. Using published data in Mendelian randomization: a blueprint for efficient identification of causal risk factors. Eur J Epidemiol 2015;30:543–52. 25773750 10.1007/s10654-015-0011-z PMC4516908

[7] Pierce BL, Burgess S. Efficient Design for Mendelian Randomization Studies: Subsample and 2-Sample Instrumental Variable Estimators. Am J Epidemiol 2013;178:1177–84. 23863760 10.1093/aje/kwt084 PMC3783091

[8] Brumpton B, Sanderson E, Hartwig FP et al Within-family studies for Mendelian randomization: avoiding dynastic, assortative mating, and population stratification biases. Nat Commun 2020;11:3519. 32665587 10.1038/s41467-020-17117-4 PMC7360778

[9] Lipsitch M, Tchetgen ET, Cohen T. Negative controls: a tool for detecting confounding and bias in observational studies. Epidemiology 2010;21:383–88. 20335814 10.1097/EDE.0b013e3181d61eeb PMC3053408

[10] Lawlor DA, Tilling K, Davey Smith G. Triangulation in aetiological epidemiology. Int J Epidemiol 2016;45:1866–86. 28108528 10.1093/ije/dyw314 PMC5841843

[11] Arnold BF, Ercumen A, Benjamin-Chung J, Colford JM Jr. Brief report: negative controls to detect selection bias and measurement bias in epidemiologic studies. Epidemiology 2016;27:637–41. 27182642 10.1097/EDE.0000000000000504 PMC4969055

[12] Hill AB. The environment and disease: association or causation? Proc R Soc Med 1965;58:295–300. 14283879 10.1177/003591576505800503 PMC1898525

[13] Davey Smith G. Post-modern epidemiology: when methods meet matter. Am J Epidemiol 2019;188:1410–19. 30877306 10.1093/aje/kwz064 PMC6670067

[14] Bell JA, Carslake D, Wade KH et al Influence of puberty timing on adiposity and cardiometabolic traits: A Mendelian randomization study. PLOS Med 2018;15:e1002641. 30153260 10.1371/journal.pmed.1002641 PMC6112630

[15] Kwok MK, Leung GM, Schooling CM. Habitual coffee consumption and risk of type 2 diabetes, ischemic heart disease, depression and Alzheimer’s disease: a Mendelian randomization study. Sci Rep 2016;6:36500. 27845333 10.1038/srep36500 PMC5109212

[16] Gage SH, Jones HJ, Burgess S et al Assessing causality in associations between cannabis use and schizophrenia risk: a two-sample Mendelian randomization study. Psychol Med 2017;47:971–80. 27928975 10.1017/S0033291716003172 PMC5341491

[17] Hemani G, Zheng J, Elsworth B et al The MR-Base platform supports systematic causal inference across the human phenome. Elife 2018;7:e34408. 29846171 10.7554/eLife.34408 PMC5976434

[18] Cardon LR, Palmer LJ. Population stratification and spurious allelic association. Lancet 2003;361:598–604. 12598158 10.1016/S0140-6736(03)12520-2

[19] Barton N, Hermisson J, Nordborg M. Why structure matters. eLife 2019;8:e45380. 30895925 10.7554/eLife.45380 PMC6428565

[20] Price AL, Patterson NJ, Plenge RM, Weinblatt ME, Shadick NA, Reich D. Principal components analysis corrects for stratification in genome-wide association studies. Nat Genet 2006;38:904–09. 16862161 10.1038/ng1847

[21] Loh P-R, Tucker G, Bulik-Sullivan BK et al Efficient Bayesian mixed-model analysis increases association power in large cohorts. Nat Genet 2015;47:284–90. 25642633 10.1038/ng.3190 PMC4342297

[22] Yu J, Pressoir G, Briggs WH et al A unified mixed-model method for association mapping that accounts for multiple levels of relatedness. Nat Genet 2006;38:203–08. 16380716 10.1038/ng1702

[23] Zhou W, Nielsen JB, Fritsche LG et al Efficiently controlling for case-control imbalance and sample relatedness in large-scale genetic association studies. Nat Genet 2018;50:1335–41. 30104761 10.1038/s41588-018-0184-y PMC6119127

[24] Abdellaoui A, Hugh-Jones D, Yengo L et al Genetic correlates of social stratification in Great Britain. Nat Hum Behav 2019;3:1332–42. 31636407 10.1038/s41562-019-0757-5

[25] Sarmanova A, Morris T, Lawson DJ. Population stratification in GWAS meta-analysis should be standardized to the best available reference datasets. bioRxiv. 2020:2020.09.03.281568.

[26] Berg JJ, Harpak A, Sinnott-Armstrong N et al Reduced signal for polygenic adaptation of height in UK Biobank. eLife 2019;8:e39725. 30895923 10.7554/eLife.39725 PMC6428572

[27] Sohail M, Maier RM, Ganna A et al Polygenic adaptation on height is overestimated due to uncorrected stratification in genome-wide association studies. eLife 2019;8:e39702. 30895926 10.7554/eLife.39702 PMC6428571

[28] Bowden J, Davey Smith G, Haycock PC, Burgess S. Consistent estimation in Mendelian randomization with some invalid instruments using a weighted median estimator. Genet Epidemiol 2016;40:304–14. 27061298 10.1002/gepi.21965 PMC4849733

[29] Hartwig FP, Davey Smith G, Bowden J. Robust inference in summary data Mendelian randomization via the zero modal pleiotropy assumption. Int J Epidemiol 2017;46:1985–98. 29040600 10.1093/ije/dyx102 PMC5837715

[30] Bowden J, Davey Smith G, Burgess S. Mendelian randomization with invalid instruments: effect estimation and bias detection through Egger regression. Int J Epidemiol 2015;44:512–25. 26050253 10.1093/ije/dyv080 PMC4469799

[31] Sudlow C, Gallacher J, Allen N et al UK biobank: an open access resource for identifying the causes of a wide range of complex diseases of middle and old age. PloS Med 2015;12:e1001779. 25826379 10.1371/journal.pmed.1001779 PMC4380465

[32] Bycroft C, Freeman C, Petkova D et al The UK Biobank resource with deep phenotyping and genomic data. Nature 2018;562:203–09. 30305743 10.1038/s41586-018-0579-z PMC6786975

[33] Mitchell, R. (Creator), Elsworth, B. L. (Creator), Mitchell, R. (Creator), Raistrick, C. A. (Creator), Paternoster, L. (Creator), Hemani, G. (Creator), Gaunt, T. R. (Creator) (19 Feb 2019). MRC IEU UK Biobank GWAS pipeline version 2. University of Bristol. 10.5523/bris.pnoat8cxo0u52p6ynfaekeigi

[34] Hysi PG, Valdes AM, Liu F et al; International Visible Trait Genetics Consortium Genome-wide association meta-analysis of individuals of European ancestry identifies new loci explaining a substantial fraction of hair color variation and heritability. Nat Genet 2018;50:652–56. 29662168 10.1038/s41588-018-0100-5 PMC5935237

[35] Burgess S, Butterworth A, Thompson SG. Mendelian randomization analysis with multiple genetic variants using summarized data. Genet Epidemiol 2013;37:658–65. 24114802 10.1002/gepi.21758 PMC4377079

[36] Bulik-Sullivan BK, Loh P-R, Finucane HK et al; Schizophrenia Working Group of the Psychiatric Genomics Consortium. LD Score regression distinguishes confounding from polygenicity in genome-wide association studies. Nat Genet 2015;47:291–95. 25642630 10.1038/ng.3211 PMC4495769

[37] Hernán MA, Hernández-Díaz S, Robins JA. Structural approach to selection bias. Epidemiology 2004;15:615–25. 15308962 10.1097/01.ede.0000135174.63482.43

[38] Munafò MR, Tilling K, Taylor AE, Evans DM, Davey Smith G. Collider scope: when selection bias can substantially influence observed associations. Int J Epidemiol 2018;47:226–35. 29040562 10.1093/ije/dyx206 PMC5837306

[39] Hughes RA, Davies NM, Davey Smith G, Tilling K. Selection bias when estimating average treatment effects using one-sample instrumental variable analysis. Epidemiology 2019;30:350–57. 30896457 10.1097/EDE.0000000000000972 PMC6525095

[40] Cole SR, Platt RW, Schisterman EF et al Illustrating bias due to conditioning on a collider. Int J Epidemiol 2010;39:417–20. 19926667 10.1093/ije/dyp334 PMC2846442

[41] Gkatzionis A, Burgess S. Contextualizing selection bias in Mendelian randomization: how bad is it likely to be? Int J Epidemiol 2019;48:691–701. 30325422 10.1093/ije/dyy202 PMC6659463

[42] Taylor AE, Jones HJ, Sallis H et al Exploring the association of genetic factors with participation in the Avon Longitudinal Study of Parents and Children. Int J Epidemiol 2018;47:1207–16. 29800128 10.1093/ije/dyy060 PMC6124613

[43] Tchetgen Tchetgen E. The control outcome calibration approach for causal inference with unobserved confounding. Am J Epidemiol 2014;179:633–40. 24363326 10.1093/aje/kwt303 PMC3927977

[44] Sanderson E, Macdonald-Wallis C, Davey Smith G. Negative control exposure studies in the presence of measurement error: implications for attempted effect estimate calibration. Int J Epidemiol 2018;47:587–96. 29088358 10.1093/ije/dyx213 PMC5913619

