# Supplementary material for: The use of negative control outcomes in Mendelian randomization to detect potential population stratification
Source: Int J Epidemiol. 2021 Feb 11;50(4):1350–61. doi: 10.1093/ije/dyaa288 (PMC8407870; doi:10.1093/ije/dyaa288)
Supplement: dyaa288_Supplementary_Data [file dyaa288_supplementary_data.zip › ije-2020-05-0770-File002.docx]

**The use of negative control outcomes in Mendelian Randomisation to detect potential population stratification.**

**Supplementary Material**

Eleanor Sanderson*^1,2^, Tom G Richardson^1,2^, Gibran Hemani^1,2^ and George Davey Smith^1,2^

1. *MRC Integrative Epidemiology Unit at the University of Bristol, UK.*
2. *Population Health Sciences, Bristol Medical School, University of Bristol, UK.*

**Supplementary Figure S1**

We propose that for each MR study two additional MR analyses are run with a negative control outcome that is likely to be affected by population stratification. This figure gives the DAG’s for each estimation, (a) gives the main MR analysis of interest and (b) the two negative control studies. This is illustrated for an estimation of the effect of BMI on risk of coronary heart disease (CHD) where natural hair colour is used as the negative control. Genetic variants for each of these are represented by G_BMI_ and G_CHD_ respectively. In these DAG’s there is no effect of BMI or CHD on hair colour therefore any effect observed in (b) is likely to be due to population stratification.

1. DAG of the MR estimation


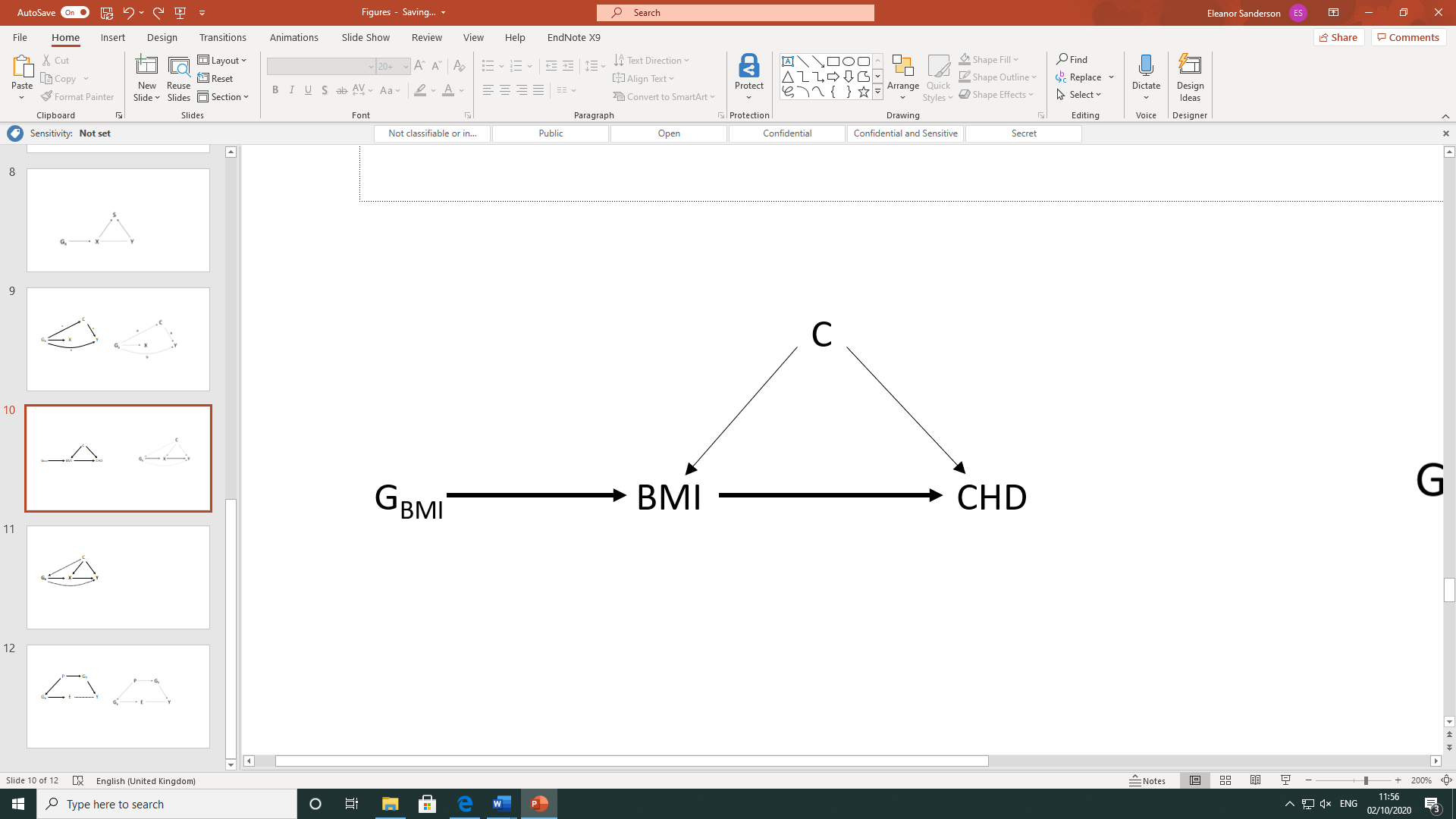


1. DAG’s for each negative control estimation to be estimated in addition to the main MR.

i.


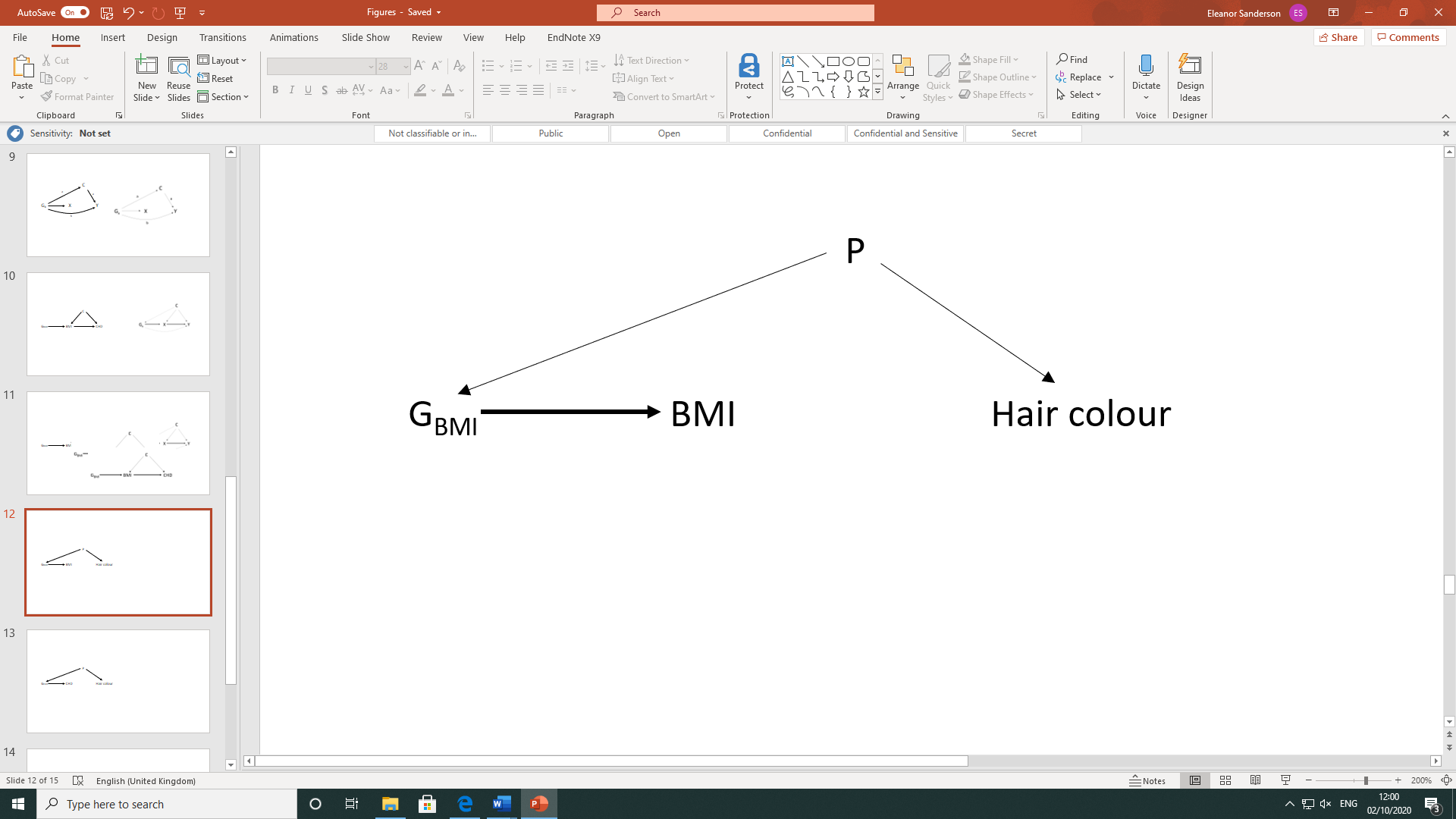


ii.


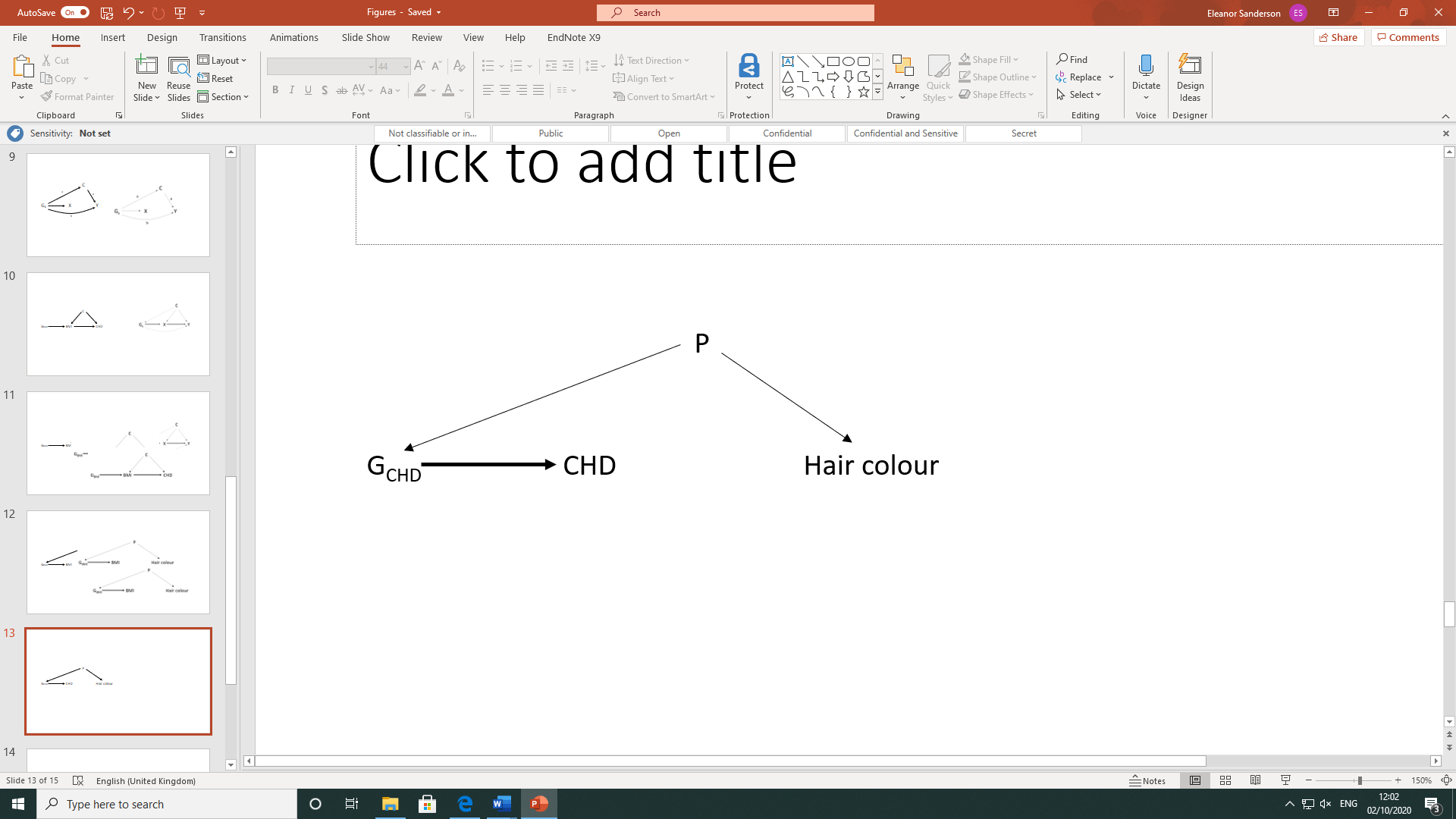


**Supplementary Table S1 –** All phenotypes included in the estimation and their estimated effect on tanning response and hair colour from a two-sample IVW estimation.

|  | **Skin tone** | | | **Hair colour** | | |  |  |  |  |  |
| --- | --- | --- | --- | --- | --- | --- | --- | --- | --- | --- | --- |
| **Exposure** | **Effect** | **Std. Error** | **P value** | **Effect** | **Std. Error** | **P value** | **No. SNPs** | **Population** | **Sample size** | **Units** | **Year** |
| Alzheimer's disease(1) | -0.004 | 0.006 | 0.5171 | 0.006 | 0.004 | 0.1582 | 20 | European | 74046 | log odds | 2013 |
| Anorexia nervosa(2) |  |  |  |  |  |  | 1 | European | 17767 | log odds | 2014 |
| Asthma(3) | -0.016 | 0.009 | 0.0711 | -0.002 | 0.005 | 0.6319 | 8 | European | 26475 | log odds | 2007 |
| Autism(4)^[[1]](#footnote-1)^ |  |  |  |  |  |  |  | European |  | log odds | 2015 |
| Bipolar disorder(5) |  |  |  |  |  |  | 4 | European | 16731 | log odds | 2011 |
| Birth length(6) |  |  |  |  |  |  | 2 | European | 28459 | SD (cm) | 2015 |
| Birth weight(7) | -0.015 | 0.021 | 0.4658 | 0.054 | 0.052 | 0.3058 | 52 | Mixed | 153781 | SD | 2016 |
| *Body Mass Index(8)* | *0.049* | *0.016* | *0.0021* | 0.011 | 0.015 | 0.4514 | *79* | *Mixed* | *339224* | *SD (kg/m^2)* | *2015* |
| Cardioembolic stroke(9) |  |  |  |  |  |  | 2 | Mixed | 21185 | NA | 2016 |
| *Celiac disease(10)* | *0.007* | *0.002* | *0.0001* | *-0.004* | *0.002* | *0.0395* | *13* | *European* | *24269* | *log odds* | *2011* |
| *Childhood obesity(11)* | *0.028* | *0.014* | *0.0400* | 0.005 | 0.010 | 0.5942 | *5* | *European* | *13848* | *log odds* | *2012* |
| Chronic kidney disease(12) |  |  |  |  |  |  | 4 | Mixed | 117165 | log odds | 2015 |
| College completion(13) |  |  |  |  |  |  | 3 | European | 95427 | log odds | 2013 |
| Coronary heart disease(14) | -0.004 | 0.007 | 0.5653 | 0.003 | 0.010 | 0.7512 | 39 | Mixed | 184305 | log odds | 2015 |
| Crohn's disease(15) | 0.011 | 0.007 | 0.1109 | 0.001 | 0.009 | 0.8884 | 122 | European | 51874 | log odds | 2015 |
| Depressive symptoms(16) |  |  |  |  |  |  | 1 | European | 161460 | SD | 2016 |
| Difference in height between adolescence and adulthood(17) |  |  |  |  |  |  | 1 | European | 9228 | SD | 2013 |
| Difference in height between childhood and adulthood(17) |  |  |  |  |  |  | 1 | European | 10799 | SD | 2013 |
| Eczema(18) | 0.002 | 0.011 | 0.8663 | 0.006 | 0.015 | 0.6908 | 12 | European | 40835 | log odds | 2014 |
| *Extreme body mass index(19)* | 0.011 | 0.006 | 0.0636 | *0.008* | *0.004* | *0.0280* | *7* | *European* | *16068* | *log odds* | *2013* |
| *Extreme height(19)* | *-0.013* | *0.006* | *0.0249* | -0.006 | 0.007 | 0.4119 | *45* | *European* | *16196* | *log odds* | *2013* |
| Extreme waist-to-hip ratio(19) |  |  |  |  |  |  | 2 | European | 10255 | log odds | 2013 |
| Gout(20) |  |  |  |  |  |  | 2 | European | 69374 | log odds | 2013 |
| *HDL cholesterol(21)* | *-0.034* | *0.016* | *0.0286* | 0.000 | 0.013 | 0.9858 | *87* | *Mixed* | *187167* | *SD (mg/dL)* | *2013* |
| Height(22) | -0.021 | 0.011 | 0.0591 | 0.002 | 0.013 | 0.8919 | 367 | European | 253288 | SD (m) | 2014 |
| *Inflammatory bowel disease(15)* | *0.009* | *0.003* | *0.0011* | -0.006 | 0.003 | 0.0794 | *63* | *European* | *34652* | *log odds* | *2015* |
| Ischaemic stroke(23) |  |  |  |  |  |  | 1 | European | 517 | log odds | 2007 |
| *LDL cholesterol(21)* | -0.002 | 0.007 | 0.7736 | *-0.020* | *0.009* | *0.0304* | *79* | *Mixed* | *173082* | *SD (mg/dL)* | *2013* |
| Lung adenocarcinoma(24) |  |  |  |  |  |  | 2 | European | 18336 | log odds | 2014 |
| Lung cancer(24) | -0.002 | 0.007 | 0.7215 | 0.009 | 0.008 | 0.2985 | 5 | European | 27209 | log odds | 2014 |
| Multiple sclerosis(25) | 0.003 | 0.003 | 0.2420 | -0.001 | 0.004 | 0.7630 | 46 | European | 38589 | log odds | 2013 |
| Myocardial infarction(14) | -0.010 | 0.009 | 0.2373 | 0.012 | 0.012 | 0.3135 | 25 | Mixed | 171875 | log odds | 2015 |
| Neuroticism(16) | 0.054 | 0.066 | 0.4100 | -0.040 | 0.074 | 0.5921 | 9 | European | 170911 | SD | 2016 |
| *Obesity class 1(19)* | *0.020* | *0.010* | *0.0380* | 0.001 | 0.008 | 0.9105 | *17* | *European* | *98697* | *log odds* | *2013* |
| *Obesity class 2(19)* | *0.011* | *0.005* | *0.0297* | *0.010* | *0.004* | *0.0097* | *11* | *European* | *72546* | *log odds* | *2013* |
| Obesity class 3(19) |  |  |  |  |  |  | 2 | European | 50364 | log odds | 2013 |
| *Overweight(19)* | *0.036* | *0.015* | *0.0181* | 0.002 | 0.011 | 0.8450 | *14* | *European* | *158855* | *log odds* | *2013* |
| Paget's disease(26) | -0.002 | 0.002 | 0.3849 | 0.002 | 0.002 | 0.3074 | 5 | European | 3440 | log odds | 2011 |
| Parkinson's disease(27) |  |  |  |  |  |  | 4 | European | 1672 | log odds | 2011 |
| Rheumatoid arthritis(28) | 0.006 | 0.007 | 0.3881 | 0.007 | 0.009 | 0.4074 | 47 | Mixed | 80799 | log odds | 2014 |
| Schizophrenia(29) | -0.004 | 0.006 | 0.4838 | -0.002 | 0.006 | 0.7681 | 71 | Mixed | 82315 | log odds | 2014 |
| Squamous cell lung cancer(24) |  |  |  |  |  |  | 4 | European | 18313 | log odds | 2014 |
| Subjective well being(16) |  |  |  |  |  |  | 1 | European | 298420 | SD | 2016 |
| *Total cholesterol(21)* | 0.003 | 0.010 | 0.7604 | *-0.028* | *0.010* | *0.0050* | *87* | *Mixed* | *187365* | *SD (mg/dL)* | *2013* |
| *Triglycerides(21)* | *0.042* | *0.013* | *0.0008* | *-0.053* | *0.015* | *0.0004* | *54* | *Mixed* | *177861* | *SD (mg/dL)* | *2013* |
| *Type 2 diabetes(30)* | -0.003 | 0.016 | 0.8646 | *-0.029* | *0.024* | *0.2346* | *36* | *European* | *69033* | *log odds* | *2012* |
| Ulcerative colitis(15) | 0.005 | 0.003 | 0.0649 | -0.003 | 0.004 | 0.4628 | 85 | European | 47745 | log odds | 2015 |
| *Waist circumference(31)* | *0.050* | *0.023* | *0.0302* | -0.035 | 0.044 | 0.4334 | *46* | *Mixed* | *224459* | *SD (cm)* | *2015* |
| Waist-to-hip ratio(31) | 0.026 | 0.019 | 0.1677 | 0.009 | 0.029 | 0.7500 | 30 | Mixed | 224459 | SD | 2015 |
| *Years of schooling(32)* | *0.074* | *0.028* | *0.0077* | *0.070* | *0.027* | *0.0095* | *70* | *European* | *293723* | *SD (years)* | *2016* |

1. Lambert JC, Ibrahim-Verbaas CA, Harold D, Naj AC, Sims R, Bellenguez C, et al. Meta-analysis of 74,046 individuals identifies 11 new susceptibility loci for Alzheimer's disease. Nature genetics. 2013;45(12):1452-8.

2. Boraska V, Franklin CS, Floyd JA, Thornton LM, Huckins LM, Southam L, et al. A genome-wide association study of anorexia nervosa. Molecular psychiatry. 2014;19(10):1085-94.

3. Moffatt MF, Gut IG, Demenais F, Strachan DP, Bouzigon E, Heath S, et al. A large-scale, consortium-based genomewide association study of asthma. The New England journal of medicine. 2010;363(13):1211-21.

4. Zhao H, Nyholt DRJHG. Gene-based analyses reveal novel genetic overlap and allelic heterogeneity across five major psychiatric disorders. 2017;136(2):263-74.

5. Group PGCBDW. Large-scale genome-wide association analysis of bipolar disorder identifies a new susceptibility locus near ODZ4. Nature genetics. 2011;43(10):977-83.

6. van der Valk RJ, Kreiner-Moller E, Kooijman MN, Guxens M, Stergiakouli E, Saaf A, et al. A novel common variant in DCST2 is associated with length in early life and height in adulthood. Human molecular genetics. 2015;24(4):1155-68.

7. Horikoshi M, Beaumont RN, Day FR, Warrington NM, Kooijman MN, Fernandez-Tajes J, et al. Genome-wide associations for birth weight and correlations with adult disease. Nature. 2016;538(7624):248-52.

8. Locke AE, Kahali B, Berndt SI, Justice AE, Pers TH, Day FR, et al. Genetic studies of body mass index yield new insights for obesity biology. Nature. 2015;518(7538):197-206.

9. Malik R, Traylor M, Pulit SL, Bevan S, Hopewell JC, Holliday EG, et al. Low-frequency and common genetic variation in ischemic stroke: The METASTROKE collaboration. Neurology. 2016;86(13):1217-26.

10. Trynka G, Hunt KA, Bockett NA, Romanos J, Mistry V, Szperl A, et al. Dense genotyping identifies and localizes multiple common and rare variant association signals in celiac disease. Nature genetics. 2011;43(12):1193-201.

11. Bradfield JP, Taal HR, Timpson NJ, Scherag A, Lecoeur C, Warrington NM, et al. A genome-wide association meta-analysis identifies new childhood obesity loci. Nature genetics. 2012;44(5):526-31.

12. Pattaro C, Teumer A, Gorski M, Chu AY, Li M, Mijatovic V, et al. Genetic associations at 53 loci highlight cell types and biological pathways relevant for kidney function. Nature communications. 2016;7:10023.

13. Rietveld CA, Medland SE, Derringer J, Yang J, Esko T, Martin NW, et al. GWAS of 126,559 individuals identifies genetic variants associated with educational attainment. Science (New York, NY). 2013;340(6139):1467-71.

14. Nikpay M, Goel A, Won HH, Hall LM, Willenborg C, Kanoni S, et al. A comprehensive 1,000 Genomes-based genome-wide association meta-analysis of coronary artery disease. Nature genetics. 2015;47(10):1121-30.

15. Liu JZ, van Sommeren S, Huang H, Ng SC, Alberts R, Takahashi A, et al. Association analyses identify 38 susceptibility loci for inflammatory bowel disease and highlight shared genetic risk across populations. Nature genetics. 2015;47(9):979-86.

16. Okbay A, Baselmans BM, De Neve JE, Turley P, Nivard MG, Fontana MA, et al. Genetic variants associated with subjective well-being, depressive symptoms, and neuroticism identified through genome-wide analyses. Nature genetics. 2016;48(6):624-33.

17. Cousminer DL, Berry DJ, Timpson NJ, Ang W, Thiering E, Byrne EM, et al. Genome-wide association and longitudinal analyses reveal genetic loci linking pubertal height growth, pubertal timing and childhood adiposity. Human molecular genetics. 2013;22(13):2735-47.

18. Paternoster L, Standl M, Waage J, Baurecht H, Hotze M, Strachan DP, et al. Multi-ancestry genome-wide association study of 21,000 cases and 95,000 controls identifies new risk loci for atopic dermatitis. Nature genetics. 2015;47(12):1449-56.

19. Berndt SI, Gustafsson S, Magi R, Ganna A, Wheeler E, Feitosa MF, et al. Genome-wide meta-analysis identifies 11 new loci for anthropometric traits and provides insights into genetic architecture. Nature genetics. 2013;45(5):501-12.

20. Kottgen A, Albrecht E, Teumer A, Vitart V, Krumsiek J, Hundertmark C, et al. Genome-wide association analyses identify 18 new loci associated with serum urate concentrations. Nature genetics. 2013;45(2):145-54.

21. Willer CJ, Schmidt EM, Sengupta S, Peloso GM, Gustafsson S, Kanoni S, et al. Discovery and refinement of loci associated with lipid levels. Nature genetics. 2013;45(11):1274-83.

22. Wood AR, Esko T, Yang J, Vedantam S, Pers TH, Gustafsson S, et al. Defining the role of common variation in the genomic and biological architecture of adult human height. Nature genetics. 2014;46(11):1173-86.

23. Matarin M, Brown WM, Scholz S, Simon-Sanchez J, Fung HC, Hernandez D, et al. A genome-wide genotyping study in patients with ischaemic stroke: initial analysis and data release. The Lancet Neurology. 2007;6(5):414-20.

24. Wang Y, McKay JD, Rafnar T, Wang Z, Timofeeva MN, Broderick P, et al. Rare variants of large effect in BRCA2 and CHEK2 affect risk of lung cancer. Nature genetics. 2014;46(7):736-41.

25. Beecham AH, Patsopoulos NA, Xifara DK, Davis MF, Kemppinen A, Cotsapas C, et al. Analysis of immune-related loci identifies 48 new susceptibility variants for multiple sclerosis. Nature genetics. 2013;45(11):1353-60.

26. Albagha OM, Wani SE, Visconti MR, Alonso N, Goodman K, Brandi ML, et al. Genome-wide association identifies three new susceptibility loci for Paget's disease of bone. Nature genetics. 2011;43(7):685-9.

27. Simon-Sanchez J, Schulte C, Bras JM, Sharma M, Gibbs JR, Berg D, et al. Genome-wide association study reveals genetic risk underlying Parkinson's disease. Nature genetics. 2009;41(12):1308-12.

28. Okada Y, Wu D, Trynka G, Raj T, Terao C, Ikari K, et al. Genetics of rheumatoid arthritis contributes to biology and drug discovery. Nature. 2014;506(7488):376-81.

29. Consortium SWGotPG. Biological insights from 108 schizophrenia-associated genetic loci. Nature. 2014;511(7510):421-7.

30. Morris AP, Voight BF, Teslovich TM, Ferreira T, Segre AV, Steinthorsdottir V, et al. Large-scale association analysis provides insights into the genetic architecture and pathophysiology of type 2 diabetes. Nature genetics. 2012;44(9):981-90.

31. Shungin D, Winkler TW, Croteau-Chonka DC, Ferreira T, Locke AE, Magi R, et al. New genetic loci link adipose and insulin biology to body fat distribution. Nature. 2015;518(7538):187-96.

32. Okbay A, Beauchamp JP, Fontana MA, Lee JJ, Pers TH, Rietveld CA, et al. Genome-wide association study identifies 74 loci associated with educational attainment. Nature. 2016;533(7604):539-42.

1. This study was dropped because the description data given in MR base for sample size did not match that given in the paper for this trait. [↑](#footnote-ref-1)
